# Full C-band wavelength-tunable, 250 MHz repetition rate mode-locked polarization-maintaining fiber laser

**DOI:** 10.1038/s41598-023-30532-z

**Published:** 2023-03-03

**Authors:** Yoon-Soo Jang, Jungjae Park, Jonghan Jin

**Affiliations:** 1 Length Group, Korea Research Institute of Standards and Science, Daejeon, 34113 Republic of Korea; 2 Major in Precision Measurement, University of Science and Technology, Daejeon, 34113 Republic of Korea

**Keywords:** Mode-locked lasers, Fibre lasers

## Abstract

We demonstrate a full C-band wavelength-tunable mode-locked fiber laser with a repetition rate of 250 MHz, representing the highest repetition rate for C-band tunable mode-locked lasers thus far to the best of our knowledge. The polarization-maintaining fiber-based Fabry–Perot cavity enables a fundamental repetition rate of 250 MHz with a semiconductor saturable absorber mirror as a mode-locker. We observed a stable and single soliton mode-locking state with wide tunability of the center wavelength from 1505 to 1561 nm by adjusting the incident angle of a bandpass filter inside the cavity. The wavelength-tunable high-repetition-rate mode-locked laser covering the full C-band is expected to be a compelling source for many frequency-comb-based applications, including high-precision optical metrology, broadband absorption spectroscopy, and broadband optical frequency synthesizers.

## Introduction

High-repetition-rate mode-locked lasers, given their unique characteristics of an ultra-short pulse, high peak power and a broad spectrum, have played key roles in numerous applications, including frequency comb generation^1^, remote timing transfer and synchronization^2^, broadband spectroscopy^3^, microwave generation^4^, length metrology^5–8^, surface metrology^9^ and observations of ultrafast phenomena^10^. In particular, fiber-based mode-locked lasers have been widely used as a practical tool given their reliability, compactness and low cost^11^.

To create a high-repetition-rate mode-locked laser, the common methods are harmonic mode-locking and shortening of the cavity length. In the former method, ensuring the stable operation of harmonic mode-locking involves many technical challenges related to super-mode noise, such as a low signal-to-noise ratio and degradation of the pulse timing jitter^12^. Contrary to harmonic mode-locking, scaling up the fundamental repetition rate while shortening the cavity length can stably generate ultra-short pulses with better spectral purity and timing jitter^13^. With regard to fiber lasers, they are commonly designed with a Fabry–Perot cavity because some components can be placed outside of the cavity^14^.

Mode-locking is commonly realized by nonlinear polarization evolution (NPE)^15^ and a real saturable absorber (SA)^16^. The NPE technique has advantages with regard to the characteristics of the pulses, such as ultra-short pulse generation and a broad spectrum. However, turn-key operation scarcely works for NPE based mode-locked lasers. Contrary to the NPE technique, SAs, which are based on saturable absorption materials and demonstrated with semiconductors^17^, carbon nanotubes^18^, graphene^19^, and 2D materials^20^, offer the advantages of turn-key operation and self-mode-locking. SAs are suitable for high-repetition-rate mode-locked lasers because they only require a small area in the cavity. SA-based mode-locked lasers are typically operated in the soliton pulse regime, where the cavity net dispersion and self-phase modulation are well balanced^21^. However, compared to NPE-based mode-locked lasers, SA-based mode-locked lasers have a broader pulse width and narrower spectral bandwidth. Typically, SA-based mode-locked lasers have pulse durations of a few hundreds of femtoseconds and spectral bandwidths of a few nanometers on the C-band^22^. To generate a broadband optical spectrum and an ultra-short pulse, nonlinear fiber-based spectral broadening with power amplification is exploited as a common method. Instead of the complicated and onerous method of nonlinear spectral broadening, tuning the center wavelength of a mode-locked laser is one feasible alternative to cover a broad spectral range via a simple approach.

**Figure 1:**
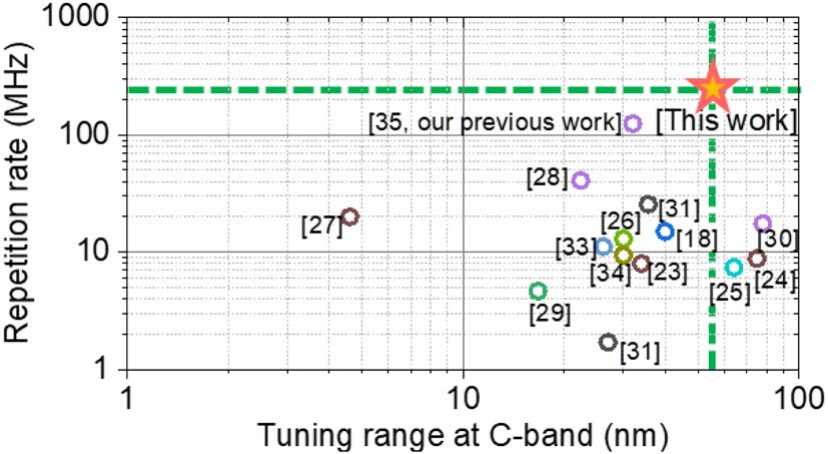
Summary of state-of-the-art C-band tunable mode-locked lasers in terms of the repetition rate and tuning range on the C-band.

Figure 1 presents an overview of C-band tunable mode-locked fiber laser results in terms of the tuning range of the center wavelength and pulse repetition rate on a logarithmic scale^18,23–35^. Wavelength-tunable mode-locked fiber lasers operating on the C-band have been demonstrated with various ways of tuning the center wavelength, including a tunable bulk filter^18,23,26,35^, an intrinsic cavity birefringence effect^24,29,33,34^, an extendable grating^25^, a 45°-tilted fiber grating^27,31^, a super-mode interference effect^28^ and a grating with a tuning aperture^30,32^. Thus far, C-band tunable mode-locked lasers have only been demonstrated at repetition rates below the range of tens of MHz, while the tunable range of the center wavelength already covers the full C-band. These types of mode-locked lasers with a low repetition rate are suitable for laser machining and high-power amplification, but their repetition rates are still too low for a majority of frequency-comb-based optical metrology applications^13,36^.

In our previous study, we demonstrated for the first time a widely wavelength-tunable mode-locked fiber laser operating at a repetition rate of more than 100 MHz^35^. The Fabry–Perot cavity-based optical design enabled a high-repetition-rate mode-locked fiber laser with full C-band tunability. In this study, we report a full C-band tunable mode-locked polarization-maintaining fiber mode-locked laser operating at a fundamental repetition rate of 250 MHz, which is the highest repetition rate for a C-band tunable mode-locked fiber laser thus far to the best of our knowledge. Such a high repetition rate makes it easy to phase-lock or to monitor the optical frequency of a continuous-wave laser. The polarization-maintaining fiber structure enables robust and turn-key operation without intra-cavity polarization adjustments and offers robust and stable mode-locking operation^13,22,37^. A semiconductor saturable absorber mirror (SESAM), commonly adopted as a well-verified, reliable, and stable mode-locker, was exploited as both an end mirror for the Fabry–Perot laser cavity and as a device to realize mode-locking with turn-key operation. A bulk optical bandpass filter was used to minimize the laser cavity length. The transmission band can easily be adjusted by changing the incidence angle of the optical band pass filter^38^. We observed a stable mode-locking state with a center wavelength from 1505 to 1561 nm. Pulse durations typically ranged from 640 fs to 2.8 ps and the spectral bandwidths (3-dB) were typically in the range of 0.9 nm to 5.1 nm. Measured output power levels exceeded 5 mW regardless of the center wavelength, making it sufficient for use in most frequency comb applications, including high-precision dimensional metrology, broadband absorption spectroscopy, and broadband optical frequency synthesizers. More importantly, it is expected that the signal-to-noise ratio in optical metrology can be improved, as the individual comb modes of the proposed mode-locked laser can have significantly more optical power than compared to the power levels offered by previous wavelength-tunable mode-locked lasers.

### Optical layout of the C-band wavelength-tunable mode-locked polarization-maintaining fiber laser

**Figure 2:**
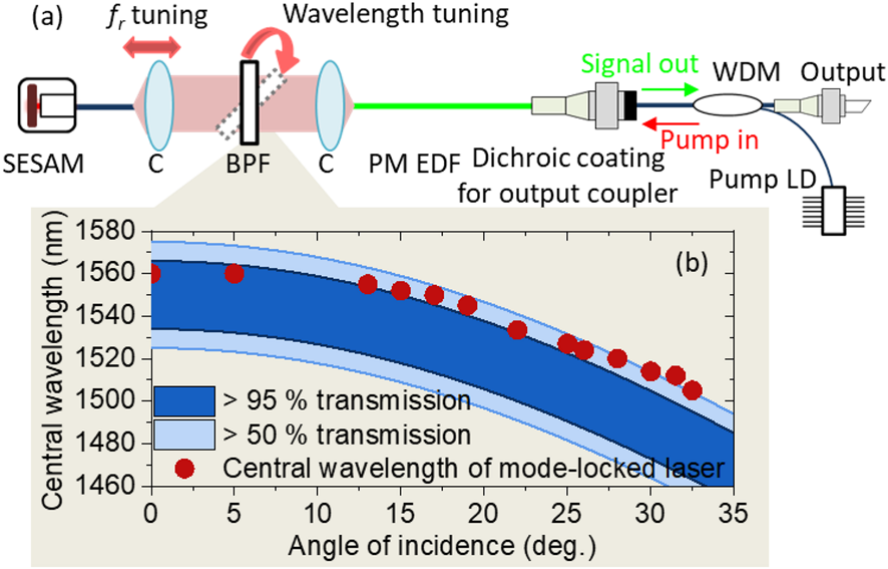
Layout of the full C-band tunable mode-locked polarization-maintaining fiber laser: (**a**) optical layout of the laser cavity of the C-band tunable mode-locked laser. C: fiber collimator. BPF: bandpass filter, WDM: wavelength multiplexer, Pump LD: pump laser diode. (**b**) Transmission curve of the bandpass filter versus the incidence angle. Red dots indicate the measured center wavelength of the mode-locked laser versus the incidence angle.

Figure 2a shows the optical layout of the proposed C-band wavelength-tunable mode-locked polarization-maintaining erbium-doped fiber laser. The design of the laser cavity is based on a fiber Fabry–Perot type of linear cavity with polarization-maintaining (PM) fiber to maintain intra-cavity polarization for robust operation. The laser cavity consists of polarization-maintaining erbium-doped fiber (PM EDF, PM-ESF-7/125, Nufern) as a gain medium, a free space part with a bulk optical bandpass filter having a 3-db bandwidth of 50 nm (7527: 1550–50 OD4, Alluxa), single-mode polarization-maintaining fiber, and the aforementioned SESAM (SAM-1550–23-2 ps, BATOP), which is a critical component for self-starting mode-locking and for the determination of the pulse characteristics^39^. The 430 mm laser cavity consists of 300 mm of PM EDF with anomalous dispersion, 60 mm of standard PM fiber and 70 mm of free space with a bulk optical band pass filter. The round-trip cavity dispersion was estimated to be -0.015 ps^2^, allowing the laser to operate in the soliton mode-locking state. The free-space part was about 70 mm and can be moved to adjust the repetition rate. The fiber-to-free-space-to-fiber coupling loss is less than 1 dB (20%). The optical bandpass filter installed in the free space part was used to tune the center wavelength of the mode-locked laser by adjusting the incidence angle. If the incidence angle of the intra-cavity light is tilted away from the normal direction (or if the incidence angle is 0°), the transmission spectrum of the optical bandpass filter will shift toward a shorter wavelength (blue shift) according to the following equation,

1 $${\lambda }_{tilt}={\lambda }_{normal}\sqrt{1-{(\frac{{n}_{o}}{{n}_{eff}}\mathrm{sin}\theta )}^{2}}$$

where *λ*_tilt_ is the transmitted wavelength when the optical bandpass filter is tilted at an incidence angle of *θ*, *λ*_normal_ is the transmitted wavelength when the optical bandpass filter is normal to the incoming light, *n*_o_ is the refractive index of the incident medium, and *n*_eff_ is the effective refractive index of the optical bandpass filter. Figure 2b shows numerical simulation results of the transmission curve of the optical bandpass filter used in this study versus the incidence angle. Also, the center wavelengths of the mode-locked laser were compared to the transmission curve of the optical bandpass filter versus the incidence angle. These results will be described in more detail in the following section.

The end mirrors of the laser cavity consisted of a dichroic coating at the FC/PC connector and the SESAM. Both end mirrors worked multi-functionally to reduce the number of optical components inside the laser cavity and minimize the length of the laser cavity. The dichroic coating at the end of the polarization-maintaining erbium-doped fiber was designed with 90% reflectance and 10% transmittance at a wavelength of 1550 nm and with 100% transmittance at a wavelength of 980 nm. The dichroic coating acted as an output coupler at a wavelength of 1550 nm but was transparent at a wavelength of 980 nm for optical pumping. The pumping laser with a wavelength of 980 nm was incident outside of the laser cavity from the PM fiber wavelength multiplexer (WDM) and the output signal of the mode-locked laser was emitted from the dichroic coating as an output coupler. The SESAM, which has a 14% modulation depth, 9% non-saturable loss and a 2 ps relaxation time constant, was butt-coupled to the FC/PC connector of the standard PM fiber and acted as both a mode-locker and an end mirror. In this work, we propose an optical layout without fiber fusion splicing despite the coupling loss, making this design beneficial for assembly and maintenance.

### Characteristics of the C-band wavelength-tunable mode-locked polarization-maintaining fiber laser

**Figure 3:**
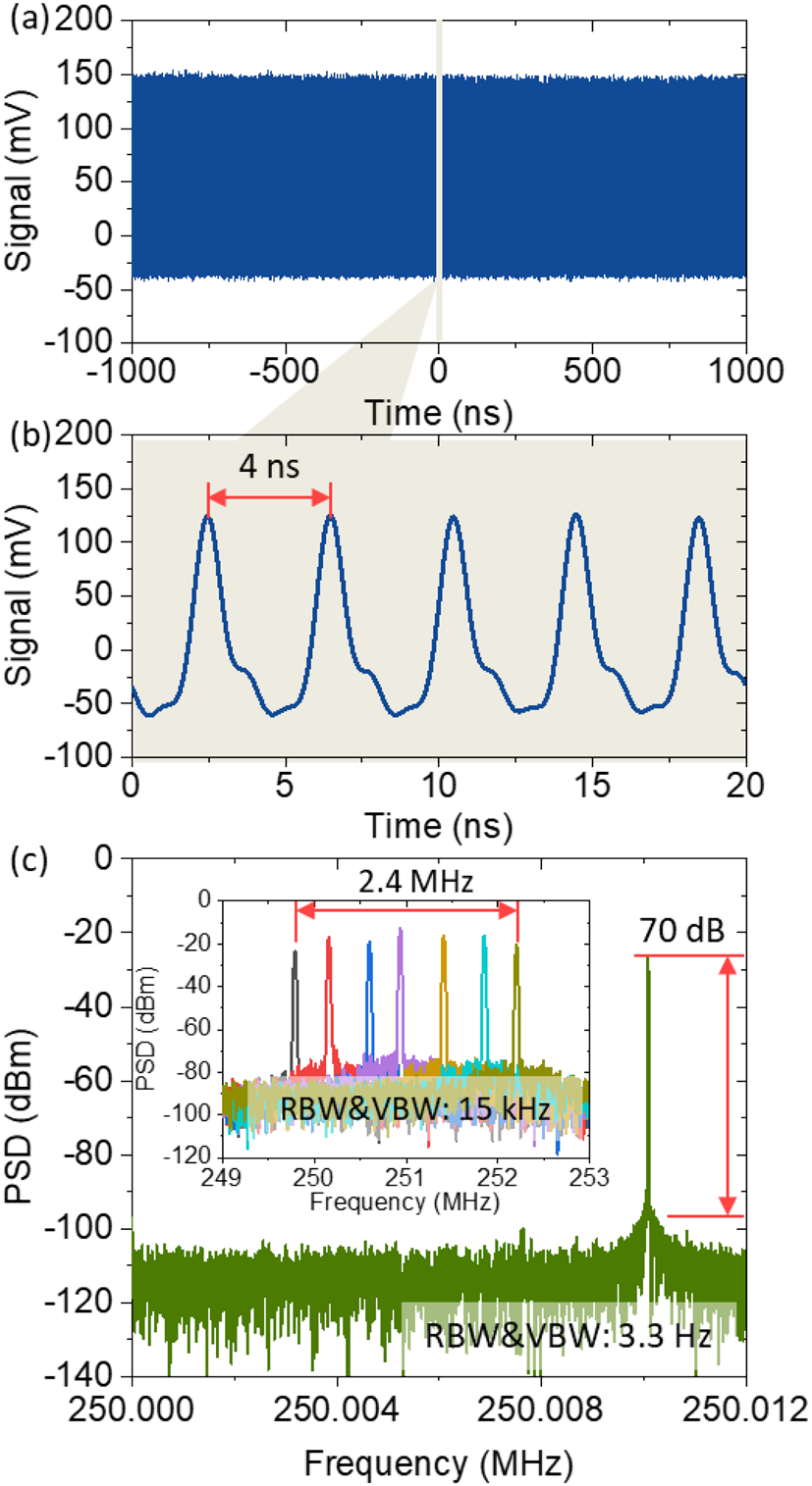
RF domain characteristics of the C-band tunable mode-locked laser: (**a**) output pulse trains over 2000 ns. (**b**) Zoomed-in view of Fig. 2a for 20 ns. (**c**) RF spectrum at the fundamental repetition rate with a resolution bandwidth and video bandwidth of 3.3 Hz. The inset shows the tunability of the repetition rate from 249.8 to 252.2 MHz with a resolution bandwidth and video bandwidth of 15 kHz.

At shorter center wavelengths (1505–1524 nm), mode-locking was typically self-starting at a pumping power of 600 mW, resulting in output power of around 10 mW. On the other hand, at longer wavelengths (1530–1561 nm), pumping power of only 400 mW could lead to self-starting with output power of around 5 mW. When mode-locking starts, the proposed mode-locked laser emits femtosecond pulses with a temporal spacing of 4 ns, corresponding to a repetition rate of 250 MHz, as shown in Fig. 3a and b. Stable pulse trains of the mode-locked laser were measured for more than 2000 ns without notable amplitude fluctuations, and a single pulse was clearly recorded, as shown in the zoomed-in view in the figure. Figure 3c shows the RF spectrum at a fundamental repetition rate of 250 MHz with both the resolution and video bandwidth set to 3.3 Hz. We measured a signal-to-noise ratio of 70 dB at a fundamental repetition rate of 250 MHz. We also measured the RF spectrum during repetition rate tuning from 249.8 to 252.2 MHz, as shown in the inset of Fig. 3c.

**Figure 4:**
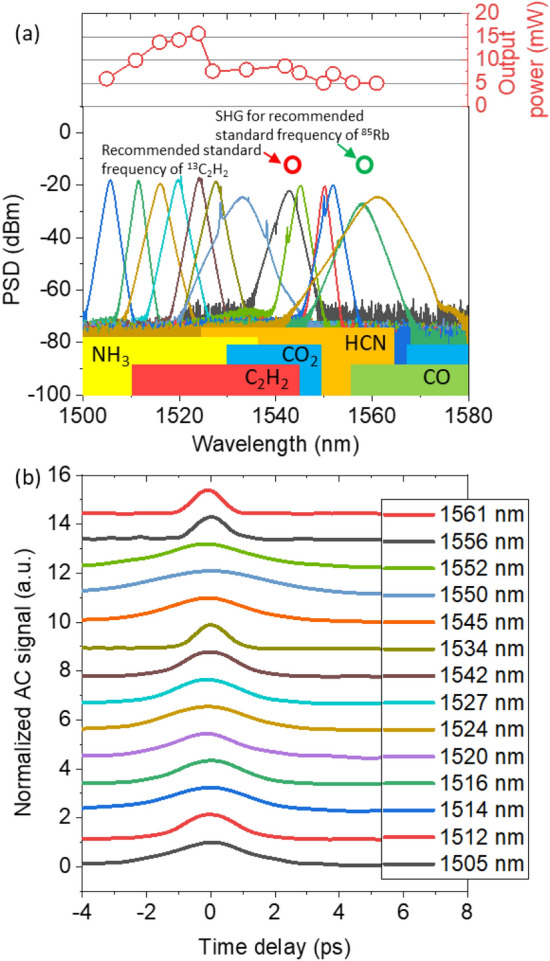
Optical characteristics of the C-band tunable mode-locked laser: (**a**) Spectral characteristics measured by an optical spectrum analyzer from 1500 to 1580 nm. The upper part shows the output power at the moment of self-starting, or at the minimum pump power. The lower part shows several important absorption lines on the C-band wavelength. SHG: second harmonic generation (**b**) Optical autocorrelation measurement for evaluating the pulse durations.

The optical characteristics of the mode-locked laser are described in Fig. 4. Figure 4a shows the measurement results of the optical spectrums and the output power outputs when tuning the center wavelength from 1505 to 1561 nm, which fully covers the C-band (1530–1565 nm). Note that this also covers the absorption line of many greenhouse gases, including NH_3_, C_2_H_2_, CO_2_, HCN and CO. Moreover, it can cover the absorption line of ^13^C_0_H_2_ near 1542 nm and ^85^Rb near 778 nm by means of second harmonic generation, which are the recommended absorption lines for the length standard by BIPM (Bureau international des poids et mesures)^40^. Importantly, the individual comb modes of the proposed mode-locked laser can have much more optical power than previous wavelength-tunable mode-locked lasers due to the high-repetition rate, which allows a better signal-to-noise ratio. At an incidence angle of 0°, the mode-locked laser typically showed a center wavelength of 1561 nm, a 3-dB spectral bandwidth of 5.1 nm, a pulse duration (fitted to the Sech^2^ curve) of 640 fs and output power of 5.1 mW, as shown in Fig. 4a and b. As the transmission spectrum of the optical bandpass filter was shifted toward a shorter wavelength by increasing the incidence angle, the center wavelength of the mode-locked laser was also shifted toward a shorter wavelength, as shown in Figs. 2b and 4a. We observed the deterministic generation of the mode-locked laser simply by tilting the incidence angle of the intra-cavity light. Note that the optical bandpass filter also had an influence on a suppression of Kelly sidebands, despite the fact that the bandwidth of the optical bandpass filter was notably larger than the spectral bandwidth of the mode-locked laser^41^. For stable mode-locking states, the output power typically ranged from 5 to 9 mW, except for a certain regime, in this case 1516 nm to 1524 nm. The gain of the Er-doped fiber was low at shorter wavelengths of 1505 nm to 1524 nm^42^. When increasing the wavelength, the output power was gradually increased from 6 to 15 mw due to the higher gain.

**Figure 5:**
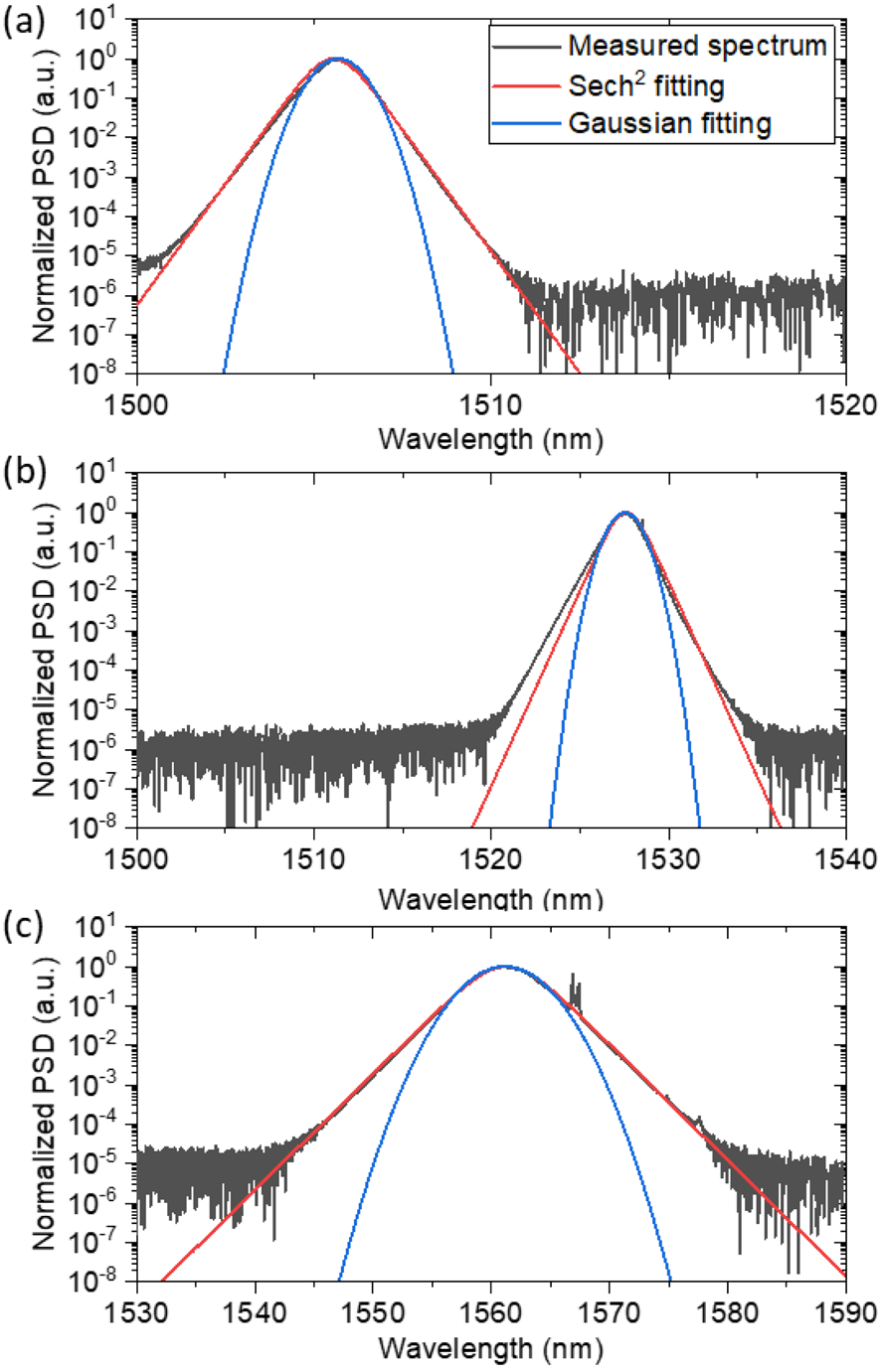
| Curve fittings of some examples of the optical spectrum to Gaussian and Sech^2^ curves: Curve fitting results for the center wavelengths of (**a**) 1505 nm, (**b**) 1527 nm, and (**c**) 1561 nm.

The optical spectrums in the entire range of the center wavelength were well fitted to the Sech^2^ curve, indicative of typical soliton pulses. Figure 5 shows examples of the optical spectrum (gray color) with Sech^2^ curve fitting (red color) and Gaussian curve fitting (blue color) for comparison. As shown in Fig. 5a–c, the measured spectrums of the mode-locked laser were well matched with both the Sech^2^ curve and the Gaussian curve near the center wavelengths. However, far from the center wavelength, the measured spectrums were well matched not to the Gaussian curve but to only the Sech^2^ curve. This clearly shows that the proposed laser operated in the soliton mode-locking regime.

**Figure 6:**
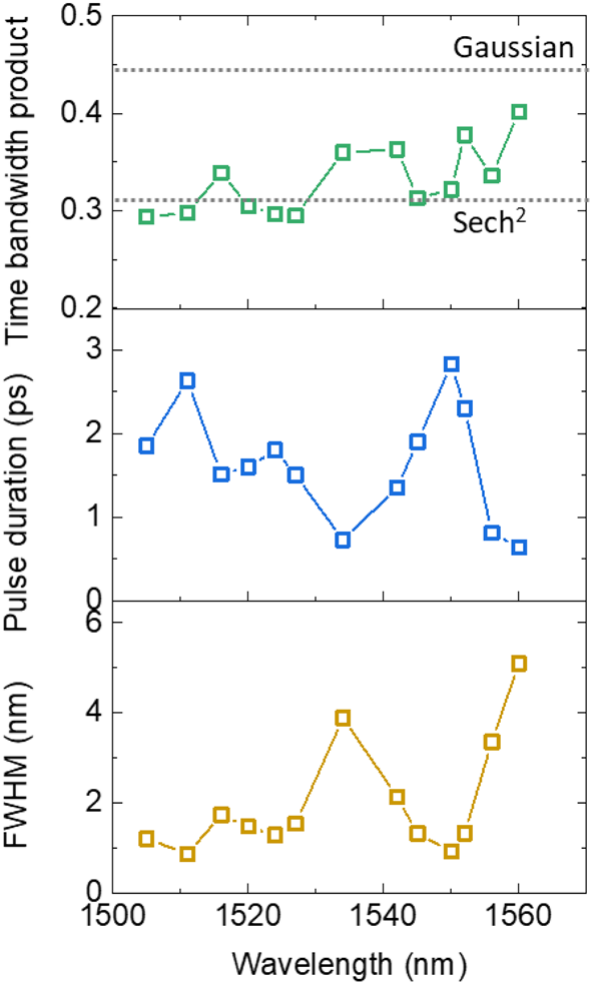
Summary of the optical characteristics of the C-band tunable mode-locked laser. The upper section, middle section and lower section show the time bandwidth product, pulse duration, and optical 3-dB bandwidth, respectively.

Figure 6 quantitatively presents in more detail the time bandwidth product, the pulse duration, and the 3-dB spectral bandwidth of the optical spectrum for each center wavelength. Pulse durations typically ranged from 640 fs to 2.8 ps assuming a Sech^2^ pulse shape, while the 3-dB spectral bandwidths ranged from 0.9 to 5.1 nm. However, they do not seem to have dependency on the center wavelength. The time bandwidth product, defined as the product of the temporal and spectral, were typically close to 0.315, corresponding to transform-limited Sech^2^ pulses. For some center wavelengths, time bandwidth products exceeding 0.315 were observed, as reported in previous studies^18,23,30^, likely caused by wavelength-dependent intracavity conditions such as cavity loss, gain, dispersion and SESAM.

## Conclusion

In this article, we proposed and demonstrated a Fabry–Perot linear cavity-based C-band wavelength-tunable mode-locked polarization-maintaining fiber laser. With a modified laser cavity design using the bulk bandpass filter in our previous study, we dramatically scaled the repetition rate up from 125 MHz of our previous study^35^ to 250 MHz, which is the highest repetition rate for a C-band tunable mode-locked laser thus far to the best of our knowledge. A wide wavelength-tunable range of 1505 nm to 1561 nm was successfully realized with turn-key operation of soliton mode-locking merely by adjusting the incidence angle of the intra-cavity light in the cavity. This simple layout enables the proposed laser to be stably and robustly operated in actual cases. The proposed laser has a repetition rate of 250 MHz, which is good enough to be exploited in most high-precision frequency comb applications, such as high-precision optical dimensional metrology, broadband spectroscopy, and high-capacity optical communications. More importantly, unlike conventional combs, the remarkably high optical power of the individual comb modes is expected to allow an improvement of the signal-to-noise ratio, thus enabling tasks that were previously difficult to perform in various applications.

## Data Availability

The datasets used and/or analyzed during the current study available from the corresponding author on reasonable request.
